# Supplementary material for: Whole-genome sequencing of chronic lymphocytic leukemia identifies subgroups with distinct biological and clinical features
Source: Nat Genet. 2022 Nov 4;54(11):1675–89. doi: 10.1038/s41588-022-01211-y (PMC9649442; doi:10.1038/s41588-022-01211-y)
Supplement: Supplementary file 1 — Supplementary Methods, Methodology, Figs. 1–7 and individual consortia authors. [file 41588_2022_1211_MOESM1_ESM.pdf]

nature genetics

Article https://doi.org/10.1038/s41588-022-01211-y

Whole-genome sequencing of chronic lymphocytic leukemia identifies subgroups with distinct biological and clinical features

In the format provided by the authors and unedited

Supplementary information for

Robbe et al. Whole-genome sequencing of chronic lymphocytic leukemia identifies subgroups with distinct biological and clinical features

Supplementary Note

Cancer cell fraction calculation

For each high-confidence CNA, the appropriately filtered, debiased and normalised median coverage R of the genomic region of the CNA (as reported by Canvas) is modelled using the following expression:

$$R = X_{ref} \cdot (1-\rho) + X_{ref} \cdot \rho \cdot (1-CCF) + X \cdot \rho \cdot CCF$$

where CCF is the cancer cell fraction for the CNA, X is the estimated copy number, X_{ref} is the copy number of the same genomic region in the normal tissue (here taken equal to 2 in all cases), and ρ is the tumour fraction in the sample. From this, it follows that an estimate $\tilde{\varphi}$ of the CCF for each CNA can be calculated as the ratio $\tilde{\varphi} = (R - X_{ref})/\left(\rho \cdot (X - X_{ref})\right)$. For samples with more than 10 copy number aberrations, we further applied a clustering step, which is typically conducted in clonal deconvolution analyses. This is because CCF values tend to aggregate in groups, which reflects the fact that tumours typically consist of homogeneous cell populations, known as *clones*. Specifically, we used mclust[1] v5.4.9 (Gaussian Mixture Modelling for Model-Based Clustering, Classification, and Density Estimation) to group the logit-transformed CCF values calculated in the previous step in an optimal number of clusters in each sample. For each CNA in a particular sample, the final estimate of the cancer cell fraction was calculated using the expression $\varphi = \left(1 + e^{-\psi}\right)^{-1}$, where $\psi = \sum_{i=1}^{K} w_i \bar{\psi}_i$. In the previous expression, K is the optimal number of estimated clusters, w_i is the probability that the CNA comes from cluster i, and $\bar{\psi}_i$ is the centre of cluster i, as reported by mclust.

For each mutation, the following relation holds between the variant allele fraction (VAF) and the cancer cell fraction (CCF) values (see Vavoulis et al. 2021[2] for a detailed derivation from first principles and references therein):

$$VAF = \frac{x \cdot \rho \cdot CCF}{X_{ref} \cdot (1-\rho) + X_{ref} \cdot \rho \cdot (1-CCF) + X \cdot \rho \cdot CCF}$$

where ρ is the tumour fraction in the sample, X is the total copy number at the locus of the SNV, x is the minor allele count at the same locus, and X_{ref} is the copy number at the same locus in the normal tissue (here taken equal to 2 in all cases). Based on this expression, rough CCF estimates for each SNV were calculated in each sample as $\tilde{\phi} = 2 \cdot VAF/(\rho \cdot x - \rho \cdot (X-2) \cdot VAF)$. In a second stage, the logit-transformed values of $\tilde{\phi}$ were clustered in each sample using mclust and final estimates were calculated using the same approach as for the copy number aberrations (see previous paragraph).

Coding variant annotations

Variants were first annotated against protein-coding sequences, non-coding gene sequences as well as splice sites and UTRs defined as per Ensembl VEP GRCh38 release 89.4. 5bp padding was added to account for splice variants at intro-exon boundaries. UTRs overlapping with protein-coding sequences were subtracted. Variants in exons of coding-genes (except Ig loci, and those mapping to chrY and chrM) were considered functional if they were predicted by Ensembl VEP to lead to aberrant splicing, protein truncation or deleterious amino acid substitution.

Non-coding variant annotations

Variants outside coding regions were annotated against non-coding regulatory elements (RE) datasets (**Fig. 4a**). All promoter regions were defined as +/- 250bp of GENCODE v29 transcription start sites. In addition, these REs were further complemented using previous available ChIP-seq data and chromatin state segmentation of primary CLL samples[3]. More specifically, these REs were defined by first intersecting previously identified H3K27ac peaks and open chromatin regions defined by ATAC-seq (derived from 104 and 106 primary CLL cases, respectively)[3]. To consider potential differences related to IGHV status, we performed a differential analysis between m-IGHV and u-IGHV CLL cases using H3K27ac and ATAC-seq peaks in 39 u-IGHV and 63 m-IGHV samples for H3K27ac ChIP-seq data, and 38 u-IGHV and 66 m-IGHV cases for ATAC-seq data. These differential analyses allowed us to precisely classify active/open chromatin regions in (1) significantly higher in u-IGHV CLL, (2) significantly higher in m-IGHV CLL and (3) no significantly different between u- and m-IGHV CLL, and therefore named "CLL". Mutations were then annotated using this classification allowing us to identify potential enrichments in particular IGHV subgroups.

Next, mutations were further annotated using genome-wide segmentations of 7 previously published CLL samples (5 mutated and 2 unmutated IGHV cases) with available ChIP-seq data of six non-redundant histone marks including H3K4me3, H3K4me1, H3K27ac, H3K36me3, H3K27me3 and H3K9me3. The segmentation was done with the chromHMM software[4] as previously described[3] and gave rise to 12 chromatin states (chmm). These 12 chmm were ActProm (active promoter, with H3K27ac and H3K4me3), WkProm (weak promoter, with H3K4me1 and H3K4me3), PoisProm (poised promoter, with H3K27me3, H3K4me1 and H3K4me3), StrEnh1 (strong enhancer 1, with H3K27ac, H3K4me1 and H3K4me3), StrEnh2 (strong enhancer 2, with H3K27ac and H3K4me1), WkEnh (weak enhancer, with H3K4me1), TxnTrans (transcription transition, with H3K36me3, H3K27ac and H3K4me1), TxnElong (transcription elongation, with H3K36me3), WkTxn (weak transcription, with low H3K36me3), H3K9me3 (H3K9me3-marked repressed heterochromatin), H3K27me3 (H3K27me3-marked repressed heterochromatin) and Het;LowSign (low-signal heterochromatin, with the absence of all six histone marks). Of note, as our annotations of non-coding variants were based on CLL samples from different cohorts than the WGS cohort, regions with the same chromatin state in at least 2 samples were used.

All non-coding variants were annotated with the chmm of the region they occurred in according to five grouped functional states:

1. Heterochromatin/silenced (Het;LowSign and H3K9me3 chmm).
2. H3K27me3 repressed (poised promoters and H3K27me3 chmm).
3. Transcription (WkTxn, H3K36me3 and TxnTrans chmm)
4. Enhancers (WkEnh, StrEnh2).
5. Promoters (WkProm, ActProm, StrEnh1)

Variants overlapping with the chromatin states annotated as promoters and enhancers without significant genetic imprints of AID-mediated somatic hypermutation were considered for all downstream analyses. Previous work has established a cut-off of 35% of variants caused by off-target AID activity[5] in REs as highly enriched in AID signature, and thus was used in our analyses (**Supplementary table 17**). C>T/G mutations in WRC̲Y motifs and their reverse complement (canonical AID) were considered to be AID-linked. The remaining variants not overlapping with protein-coding

sequences, non-coding gene sequences, splice sites, UTRs, promoters or enhancers were annotated as non-regulatory regions.

Target genes of regulatory elements

To link REs to their potential target genes, we first selected the regions previously classified as promoters (including WkProm, ActProm, StrEnh1 chmm) and enhancers (including WkEnh, StrEnh2 chmm) in at least 2/7 of the previous CLL samples. Next, these regions were intersected with the previous ChIP-seq H3K27ac data showing at least 1 peak in all the CLL samples analysed (n=104). This intersection ensures to obtain H3K27ac peaks associated with REs. Afterwards, we calculated correlation coefficients between H3K27ac levels of all the previously identified peaks related to RE with the gene expression levels of all the surrounding genes. The surrounding genes were defined according to the gene coordinates with a 2kb upstream extension with respect their start and being in the same TAD as the interrogated RE (TADs were defined by Hi-C in GM12878[6]). This strategy allowed us to identify both proximal and distal associations between regulatory elements and their target genes. To perform the correlations, we used 74 previously available CLL samples with concomitant H3K27ac and RNA-seq[3]. For each defined RE, we calculated the Pearson correlation coefficients between H3K27ac levels and gene expression levels for all possible genes with the aforementioned restrictions. Potential target genes were considered to be those showing correlations of $r \geq 0.3$, $FDR \leq 0.05$ (FDR correction per each RE) with the RE. Correlations were only performed with protein coding and lncRNA genes defined by Ensembl Genes version 105. Annotation was retrieved using the biomaRt R package version 2.50. Preferentially, we have chosen the annotations from this strategy (named CLL dataset 1).

In the case where no target gene was identified using the previous strategy, we assigned the promoter class to a target gene based on the nearest TSS using the GENCODE database (for 7/72 promoters). For the enhancer class, we used additional publicly available datasets derived from CLL primary samples. This was the case of 16 out of 126 enhancers. Firstly, three-dimensional chromatin interactions publicly available for one CLL sample was downloaded (EGA, under accession number EGAD00001004046). We used this data to annotate genes found in the same TAD as the RE (CLL dataset 2, used for 2 enhancers). Secondly, we used a dataset containing target genes of enhancers predicted using correlation of RNA-seq expression data and H3K27ac data and ATAC-seq from 12 matched CLL samples previously defined[7] (CLL dataset 3, used for 6 enhancers). Briefly, we required a minimum of 10 reads in at least 1 sample for transcripts detected by RNA-seq to be considered. Within each TAD defined using HOMER[8] from Hi-C data of the GM12878 cell line [GEO accession: GSM1181867[6]], we performed a peak-transcript correlation (Spearman correlation). All correlations > 0.3 were selected. If the distance between the H3K27ac peak centre and the nearest TSS was <2kb the peak was classified as a promoter, if not, it was classified as an enhancer.

Annotations for each enhancer-target are specified in **Supplementary table 17**. After using these three strategies to annotate enhancers to target genes, eight enhancers did not have any predicted target genes. These were left without CLL specific annotations and were intersected with the Double Elite enhancers reported in the public database GeneHancer[9].

In addition, all enhancers reported as significantly mutated were further annotated as super enhancers using previously published CLL-specific super enhancers datasets obtained from chromatin accessibility and H3K27ac ChIP-seq data using 18 CLL samples[10], available in **Supplementary table 17**.

Identification of coding driver genes

Two methodologies were used to identify coding driver genes. The first methodology included only SNVs and indels and combined results of four discovery algorithms, according to a "genes detected in at least two out of four" approach (Discovery method 1A) and a statistical approach deriving one p-value from the four p-values (Discovery method 1B). The second methodology included CNAs in addition to SNVs/indels (Discovery method 2).

For discovery method 1A, the four discovery algorithms were MutSigCV v1.41[11], OncodriveFML v2.2.0[12], OncodriveCLUSTL v1.1.1[13] and dNdScv[14]. MutSigCV was used to detect drivers on the basis of recurrence, after adjusting for factors such as replication timing, gene expression and chromatin accessibility. A coverage file across protein-coding regions for the genome assembly GRCh38 was constructed using CovGen v1.0.2 (https://github.com/tgen/CovGen) and SNPeff v4.3[15] assuming full coverage across all protein-coding regions. Gene symbols used in the input MAF, coverage file and default gene covariates file were queried against gene name synonyms in HGNC to maximise unambiguous matching between input files. OncodriveFML was used to detect drivers enriched for mutations with a high functional impact. Default parameter settings were used. Genome-wide mutation frequencies were used to model the background mutation rate. Functional impact predictions were obtained from CADD v1.5[16]. OncodriveCLUSTL was used to detect drivers containing clusters of mutations. Default parameter settings were used. The same normalised mutation frequencies were used as for OncodriveFML. dNdSCV was used to detect drivers with significant evidence of positive selection. Default parameter settings were used. Each method derived a q-value per gene, and genes were selected as drivers when $q < 0.001$ for at least two out of four algorithms.

For discovery method 1B, p-values calculated for each gene from these four methods were combined as a single p-value using two methods. First, a Z-transformed ("Stouffer") weighted test of p-values using the R package "survcomp". For each gene test per-study weights were applied according to the median ranking of driver test P-values in a set of known CLL driver genes (e.g. a driver method where the median ranking is much higher for CLL genes compared to all genes tested would be weighted proportionally greater than a method where there is little difference in the median rankings of CLL genes and all genes). Second, a weighted harmonic mean p-value was used. FDR q-values of combined results were estimated from the R package "qvalue" at a significance level of $q < 0.01$. Any genes found significant by at least one of these two methods (1A and 1B) were selected as putative CLL driver genes.

For discovery method 2, first we identified minimally overlapping regions altered by CNAs, defined as the smallest genomic regions where CNAs of four samples overlap. 74 regions were defined by this method. Second, all genes falling within these loci were analysed further to select the most probable drivers of each region. MutComFocal v1.0[17] was used to rank the genes in each region, according to the following approach:

1. We ranked the genes in each region, calculating focality and recurrence scores based on SNV, indel and CNA data. The ranked genes were categorised into tiers based on the entropy H of the posterior distribution of the scores. Genes ranked in the top 2 tiers were selected (in our dataset, this was defined as $H(P) > 2.48 \times 10^{-4}$ for "CN gain + mutation", and $> 1.01 \times 10^{-4}$ for "CN loss + mutation).

2. We refined the list of selected genes to keep only genes (a) affected by CN gains if they are suspected or proved oncogene from the literature, or (b) affected by CN loss if they are suspected or proved TSG from the literature.
3. Among these, we finally chose only genes with acquired SNVs/indels in at least 1% of the cohorts (at least 5 samples). The genes that did not satisfy this criterion were not classified as "candidate drivers'', but instead are listed in **Supplementary table 9** in the "Permissive list of genes". We provide this gene list with combined recurrence and focality scores as calculated by MutComFocal as a resource for the community for future research on CLL.

Finally, coding drivers and other findings were annotated as "candidate or previously unrecognised" if they were reported in fewer than 1% of samples in previous large-scale CLL studies[18,19], and not found in literature searches to have been associated with CLL. In addition to annotations from VEP, we also annotated the variant with the Prot2HG database to determine if a variant was within a protein domain, annotated as in a "site" (short sequence with known function) and as in a "region" (larger regions of about 100 amino acids associated with a function of the protein; more details in the original publication[20]).

Identification of non-coding driver mutations

We considered non-coding SNVs and indels falling in REs as described above and in **Fig. 4a**, excluding all regions in immunoglobulin genes and other regions previously described as false-positives due to sequencing or mapping artefacts[5]. Two independent approaches were used to identify non-coding hits in the CLL dataset considering both mutational burden and functional impact (**Fig. 4b**).

Firstly, we used OncodriveFML v2.2.0 and OncodriveCLUSTL v1.1.1 with default parameters to identify REs enriched for functional mutations or linear mutation hotspots respectively. *P* values were corrected for multiple testing using the Benjamini-Hochberg method. Genomic elements with q-values < 0.1 in at least one out of two methods and with at least two samples mutated were retained as non-coding hits. All data are reported in **Supplementary table 17**.

Secondly, we screened for the presence of single-site hotspots (*i.e*. same nucleotide mutated), and regions with high mutational density/kataegis. Regions of kataegis were identified as previously described[11]. Briefly, mutations from all tumour samples were combined and scanned for six mutations with less than two standard deviations below the median of intermutational distance of SNVs/indels, or at least 6 variants with <1 kb intermutational distance. Regions were tested for an excess of mutations using a methodology adapted from previous work[21]. *P* values were calculated per hotspot using the binomial distribution, where the raw data (q) were the number of mutations in a region, the sample size (n) was the length of the region and the probability (p) was calculated separately to produce a local and global estimation of the expected mutation rate per bp. Local mutation rate was calculated as the total number of mutations within 10kb either side of the hotspot region divided by the length of that region. Any bases overlapping with exonic regions were excluded from the counts. Global values were calculated as the total number of mutations per feature, i.e., UTRs, promoters (i.e., ActProm, WkProm, StrongEnhancer2) or enhancers (i.e., StrngEnhancer1, WkEhnacner chmm) divided by the sum total length of that feature. The observed number of mutations (q) was assumed to follow a binomial distribution, binomial (n, p_i) for region i, with null hypothesis that the region was not excessively mutated. *P* values were corrected for multiple testing using the Benjamini-Hochberg

method. Significance was considered where both the global and the local test were below a significance threshold *FDR < 0.1.*
Single-site hotspots were defined as kataegis regions of length 1 bp, and were excluded from statistical calculations.

The 126 REs were annotated with the proportion of mutations attributed to off-target AID and APOBEC activities, which are known process described in CLL[5,22]. To do so, the proportion of mutations attributed to AID (C>T/G at WRCY, and the reverse, G>A/C at RGYW) and APOBEC (C>T/G at TCW, G>A/C WGA) activities were annotated for each genomic element reported as a non-coding hit. Previous work has established a cut-off of 35% of variants caused by off-target AID activity[5] in REs as highly enriched in AID signature, and thus was used in our analyses (**Supplementary table 17**). In addition, predicted target genes of these 126 REs (see above) were included in a non-ordered gene set enrichment analysis using the R package gprofiler2 v0.2.0[23] using the Gene Ontology (BP: biological process) and Human phenotype ontology (HPO) databases available within the package.

We used DeepHaem[24] to predict the effects of the non-coding mutations in the 72 promoters with open chromatin using 73 immune cell types with open chromatin assay data. Briefly, the model looks at 1 kb DNA sequence and calculates a probability of a DNA sequence belonging to an open chromatin site in each cell type. We use an empirical threshold to classify anything above a 20 % probability of being at least a weak open chromatin site. To assess the effect of each mutation, we predicted the probability score with and without the variant in the DNA sequence and calculated the damage by subtracting the variant probability score to the reference probability score. A positive score meant the variant reduced the chromatin accessibility, such as in the case of a TF binding site loss (loss of function). A negative damage score meant the variant increased chromatin accessibility, for example by creating a previously unrecognised transcription factor binding site. Empirical thresholds were set as an absolute damage above 0.1 and 0.05 to select candidate variants with varying levels of stringency (changes above 10 and 5%, respectively).

Patients' stratification using non-negative matrix factorisation

Data were converted to a 0/1 matrix using either presence or absence of a feature, or above or below the mean, in the case of continuous variables, such as the presence of a mutation in a gene or pathway, and other not, such as telomere lengths. Although Binomial Matrix Factorization (BMF) methods exist, the NMF has been found to have a similar feature outcome capability to the BMF[25]. Further, the offset method[26,27] of the NMF takes the additional step of removing common signatures between groups, making it particularity useful for identification of uniquely group defining features.
The number of clusters in each group to be used by the NMF was determined using a combination of rank estimation methods implemented by the NMF software[28–30] including the cophenetic correlation coefficient (See figures below).

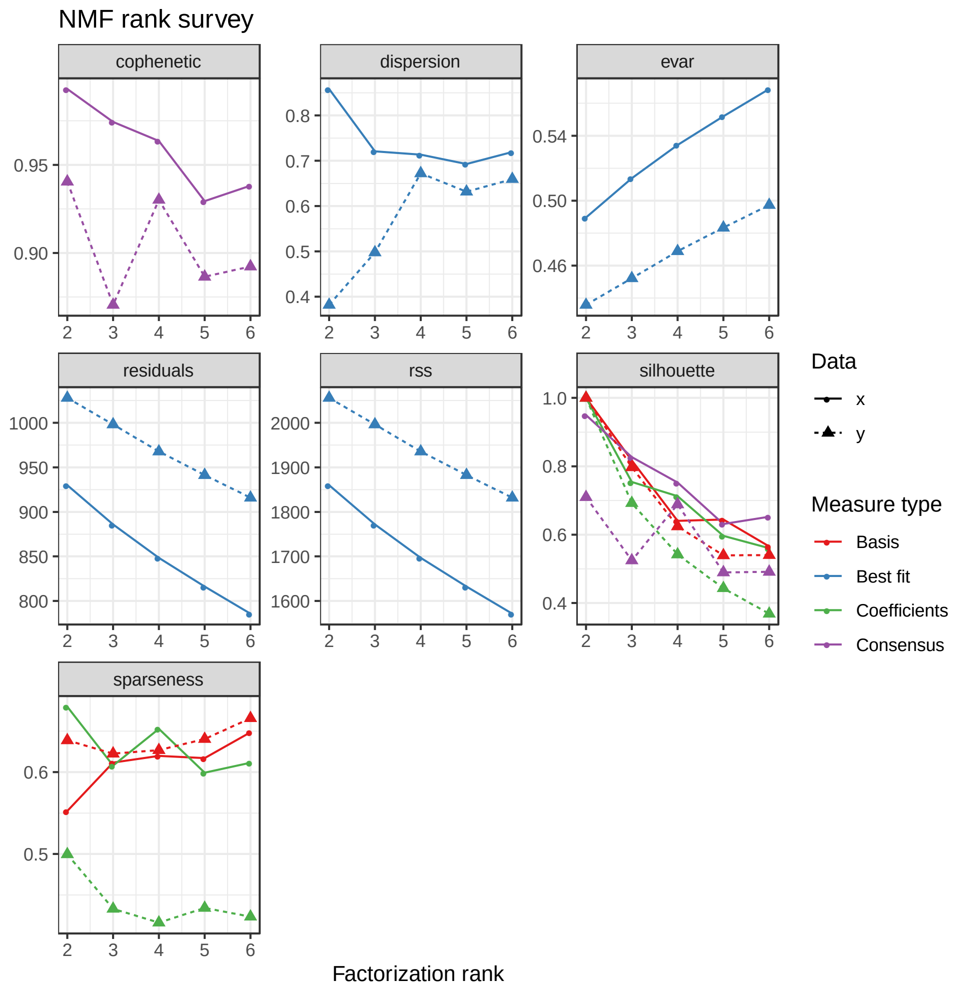

NMF factorisation plots for determining the number of clusters (k) appropriate for the NMF, m-IGHV samples (k=2)

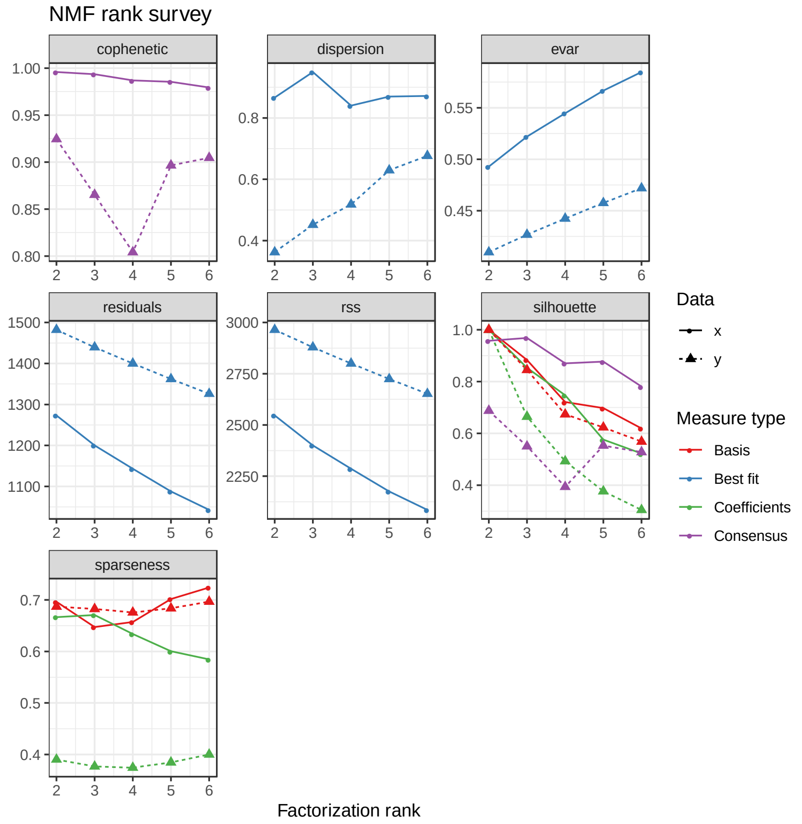

NMF factorisation plots for determining the number of clusters (k) appropriate for the NMF, u-IGHV samples (k=3).

Supplementary Figures

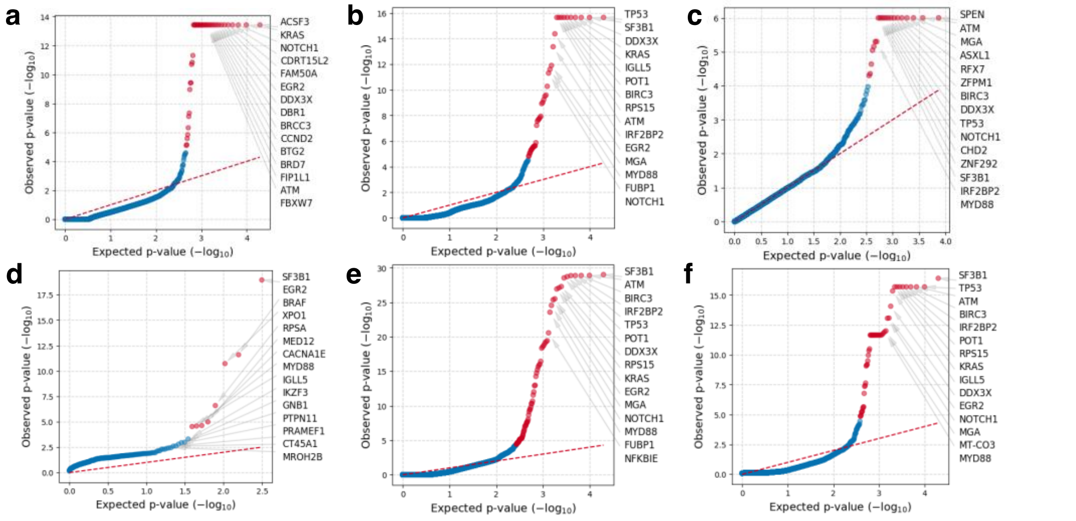

Supplementary figure 1: Somatic SNVs/indels analysis identified candidate coding drivers

a-f, Q-Q plots showing the top genes mutated discovered by (a) MutSigCV, (b) dNdSCV, **(c)** OncodriveFML, (d) OncodriveCLUSTL, (e) combined p-values using a weighted Stouffer method, (f) combined p-values using a weighted harmonic mean p-value technique. Red data points indicate q<0.1.

p-values derived by discovery algorithms were calculated using a simulation-based approach as detailed in the original publications[11–14]

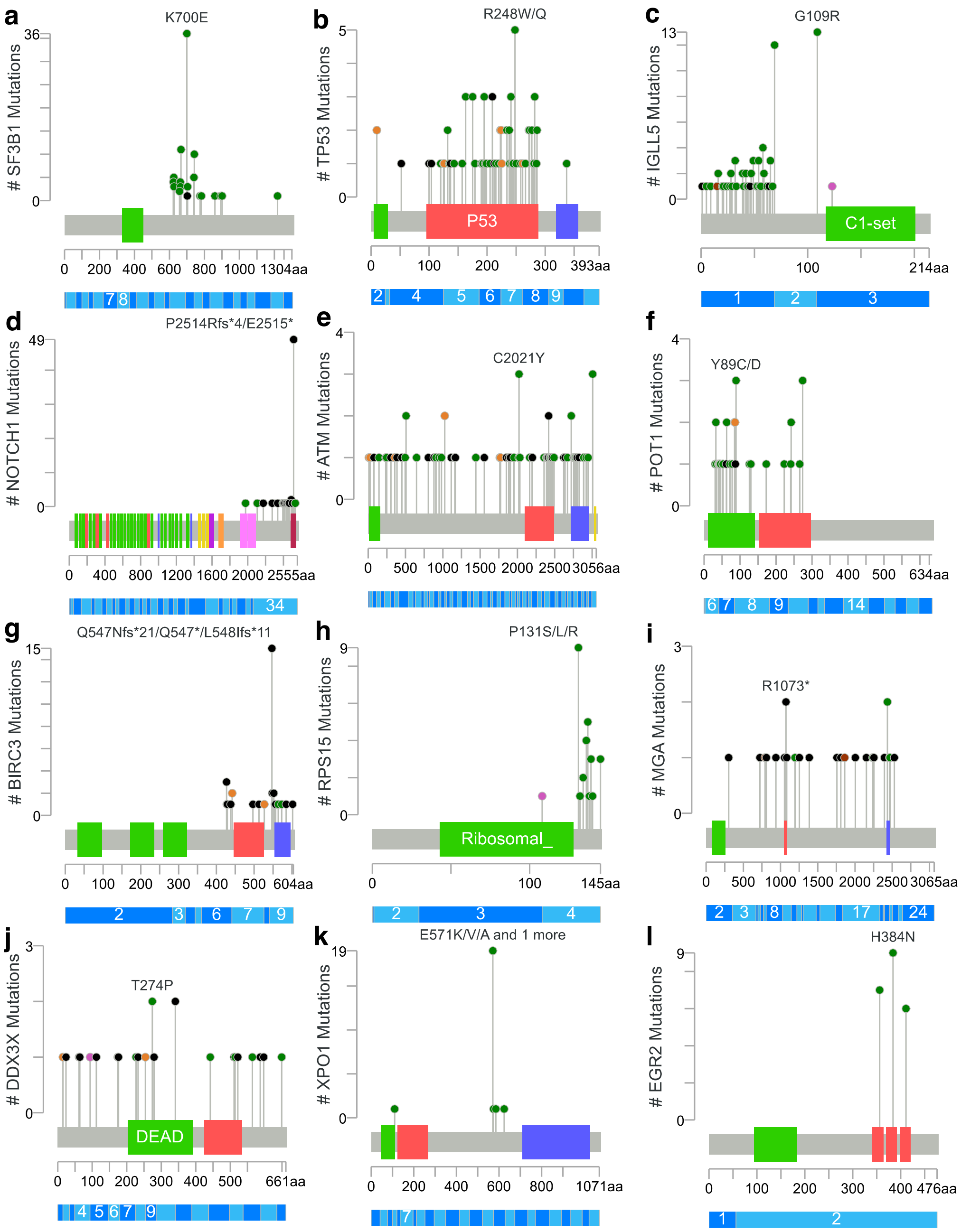

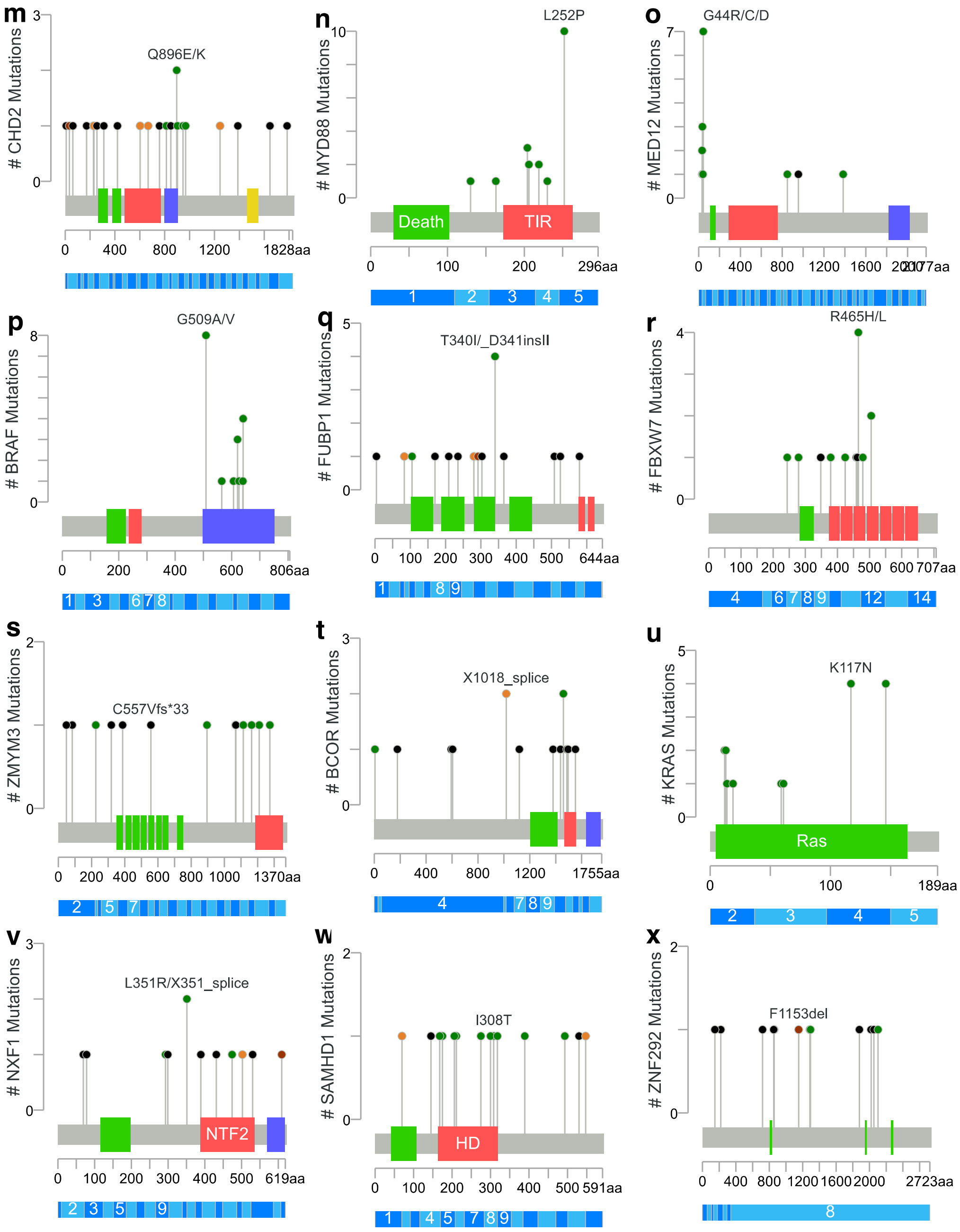
m
CHD2 Mutations
Q896E/K
0
400
800
1200
1828aa
n
MYD88 Mutations
L252P
Death
TIR
0
100
200
296aa
o
MED12 Mutations
G44R/C/D
0
400
800
1200
1600
2000
2177aa
p
BRAF Mutations
G509A/V
0
200
400
600
806aa
q
FUBP1 Mutations
T340I/_D341insII
0
100
200
300
400
500
644aa
r
FBXW7 Mutations
R465H/L
0
100
200
300
400
500
600
707aa
s
ZMYM3 Mutations
C557Vfs*33
0
200
400
600
800
1000
1370aa
t
BCOR Mutations
X1018_splice
0
400
800
1200
1755aa
u
KRAS Mutations
K117N
Ras
0
100
189aa
v
NXF1 Mutations
L351R/X351_splice
NTF2
0
100
200
300
400
500
619aa
w
SAMHD1 Mutations
I308T
HD
0
100
200
300
400
500
591aa
x
ZNF292 Mutations
F1153del
0
400
800
1200
1600
2000
2723aa

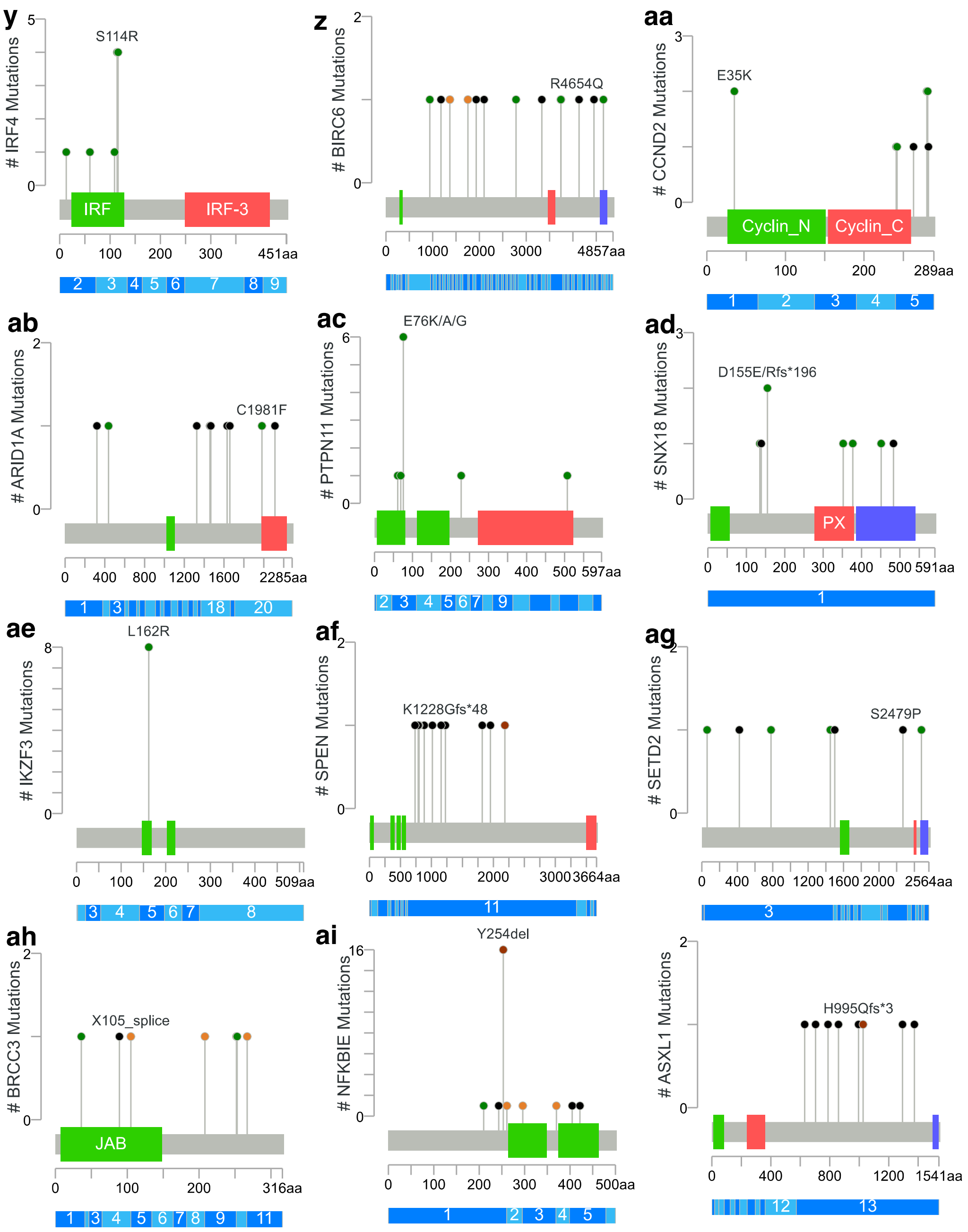

Supplementary figure 2: Distribution of variants in each of the 36 known coding drivers

a-aj, The top mutation is annotated with the amino acid change. Missense mutations are in green, truncating ones in black, inframe mutations in brown and splice side variants in orange.

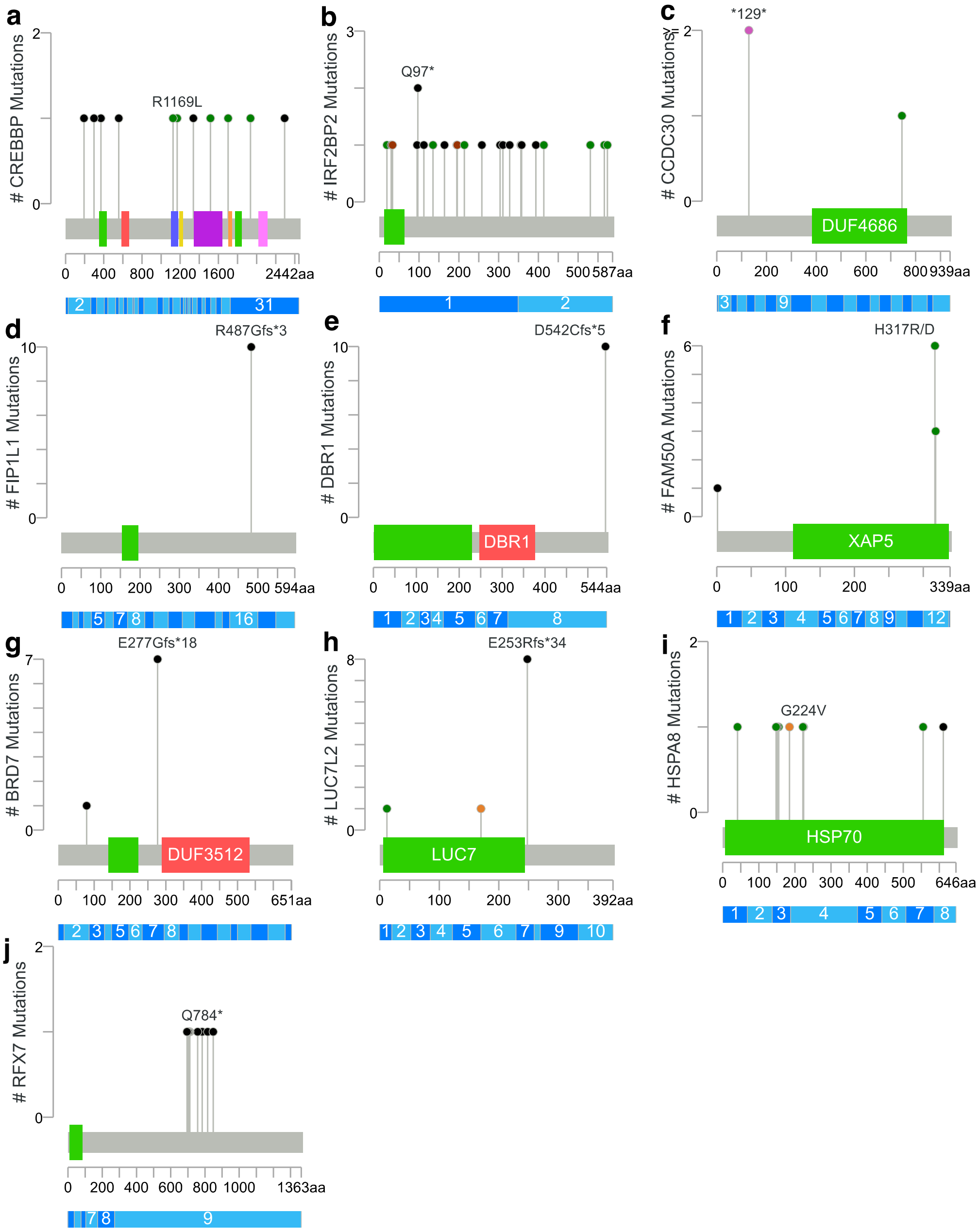

a
CREBBP Mutations
R1169L
2442aa
b
IRF2BP2 Mutations
Q97*
587aa
c
CCDC30 Mutations
129
DUF4686
939aa
d
FIP1L1 Mutations
R487Gfs*3
594aa
e
DBR1 Mutations
D542Cfs*5
DBR1
544aa
f
FAM50A Mutations
H317R/D
XAP5
339aa
g
BRD7 Mutations
E277Gfs*18
DUF3512
651aa
h
LUC7L2 Mutations
E253Rfs*34
LUC7
392aa
i
HSPA8 Mutations
G224V
HSP70
646aa
j
RFX7 Mutations
Q784*
1363aa

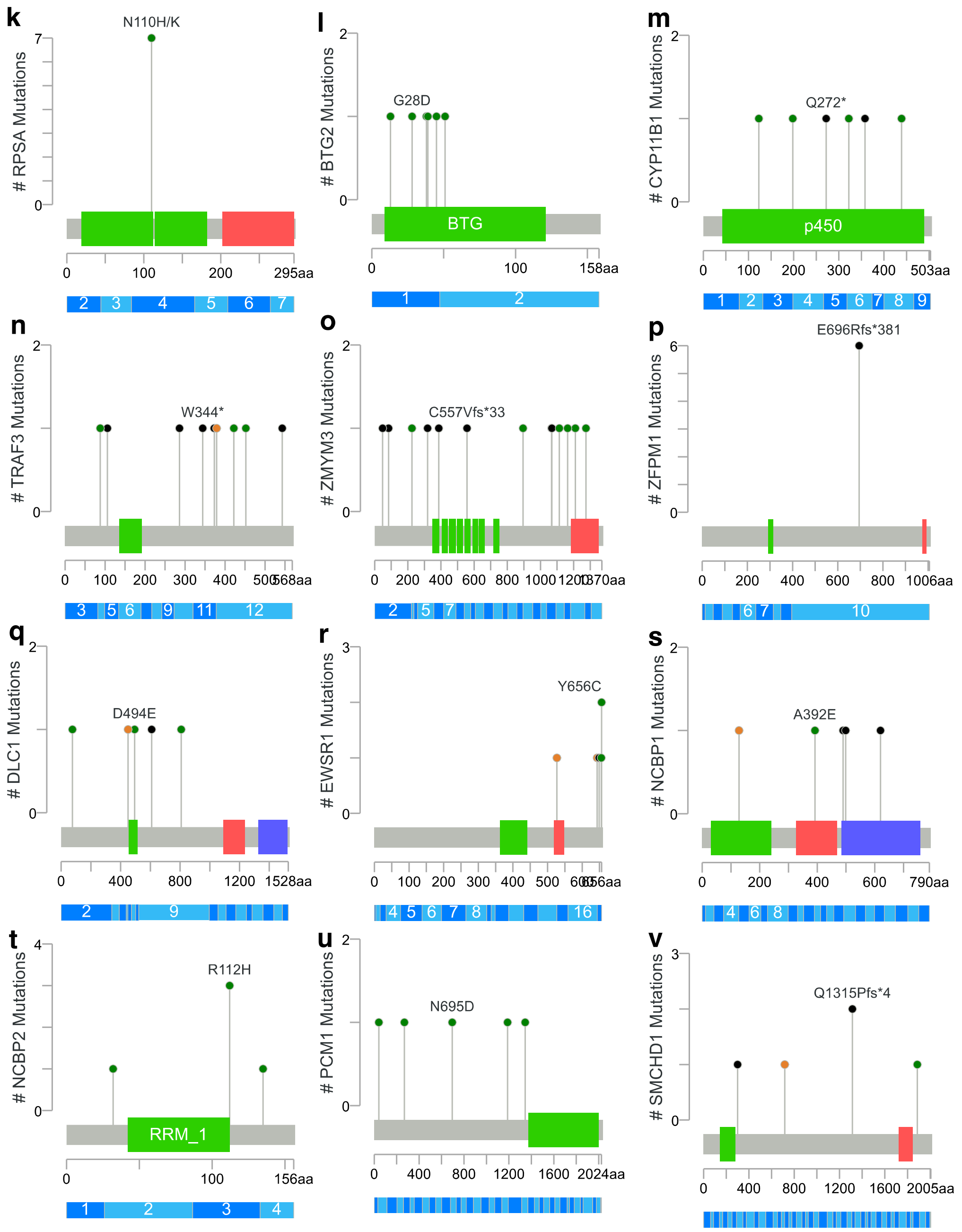

Supplementary figure 3: Distribution of variants in each of the 36 candidate coding drivers

a-v, The top mutation is annotated with the amino acid change. Missense mutations are in green, truncating ones in black, inframe mutations in brown and splice side variants in orange.

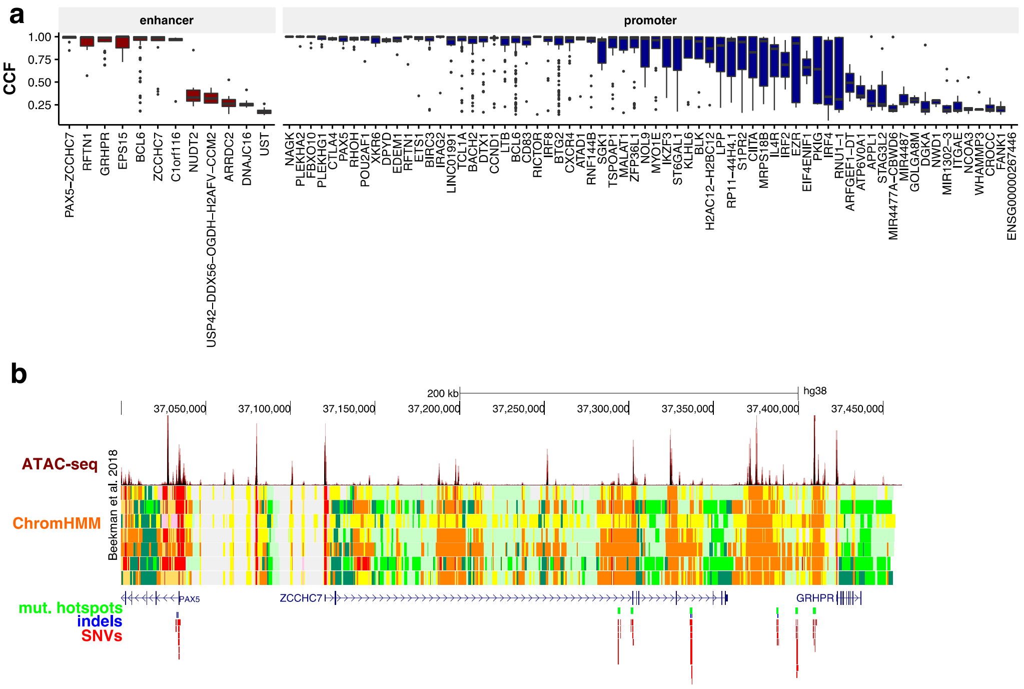

Supplementary figure 4: Exploration of significantly mutated enhancers

a, Distribution (showing the minimum and maximum values and interquantile range) of CCFs in significantly mutated enhancer and promoter regions. The number of variants represented in each boxplot is detailed in **Supplementary table 17**. **b**, Genomic view of the region containing the super-enhancer for *PAX5*. The ATAC-seq data (n=) and ChromHMM data (one sample per track) are derived from primary CLL samples and accessible from the CLL Reference Epigenome tracks in the UCSC genome browser with details about samples and legends available online[3]. The track with mutated hotspot is displaying enhancer regions that were significantly mutated: individual SNVs shown in red and indels in blue.

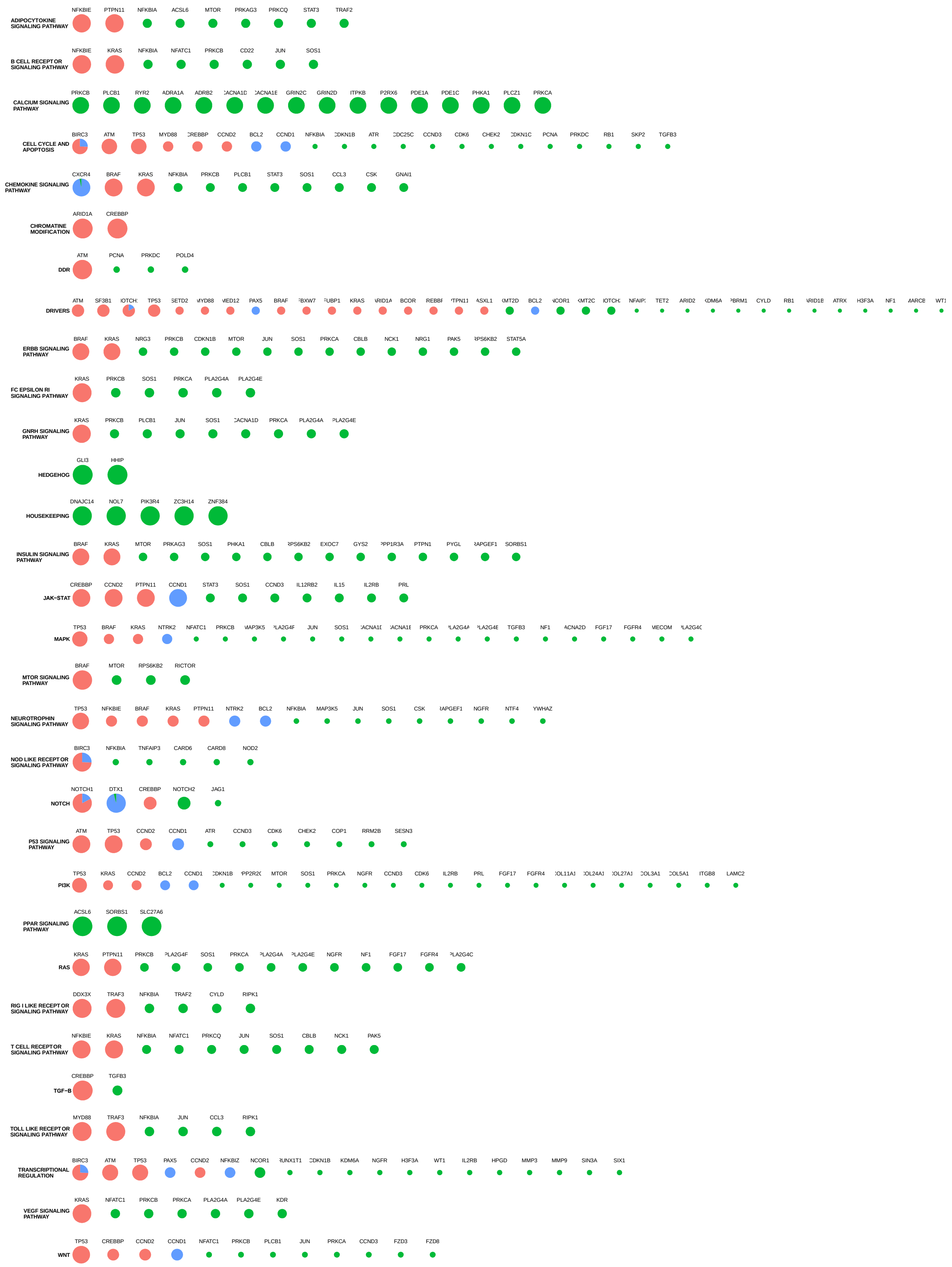

Supplementary figure 5: Mutated genes and associated regulatory elements contributing to dysregulated pathways

List of genes and associated regulatory elements recurrently mutated in each pathway. For each pathway, gene symbols are sorted from the most mutated to the least mutated. The size of the circles is proportional to the number of mutated samples and the colours denotes the type of mutations and

the type of genes affected: coding mutation in coding driver gene (red), coding mutation with high functional impact in other genes not reported as CLL driver (green), non-coding mutation in regulatory element of a gene (blue).

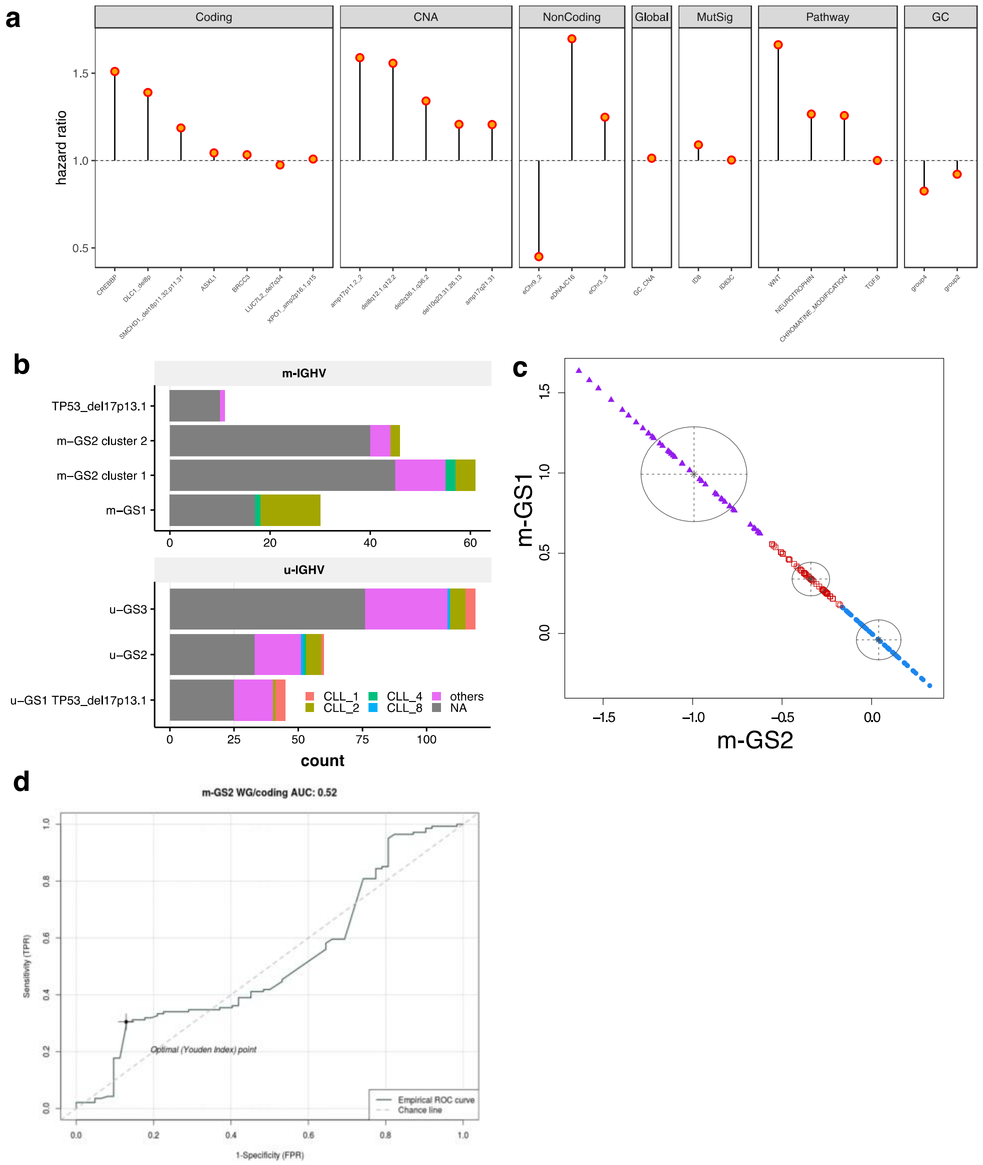

Supplementary figure 6: Integration of all genomic features defines genomic subgroups associated with clinical outcome.

a, Penalised Cox Regression performed on 186 genomic features identified independent predictors for overall-survival. Each panel shows the hazard ratios for those genomic features that jointly minimise the out-of-sample prediction error (estimated through leave-one-out cross-validation). The full list and details of each genomic feature is presented in **Supplementary Table 21**. **b**, Proportion of each subgroup defined by non-negative matrix factorization (NMF) with stereotype clusters. **c**, mclust classification of the proportion of each hypermutated genomic subgroup (m-GS1, m-GS2) calculated in each hypermutated sample using deconstructSigs. Sample proportions were logit transformed before clustering and the appropriate number of clusters approximated by examining mclust Bayesian information criterion (BIC) plots. m-GS2 into distinct two clusters (red and blue) **d**, Receiver operating characteristic (ROC) curve of m-GS2 derived from coding sequence data against m-GS2 derived from whole genome sequencing data.

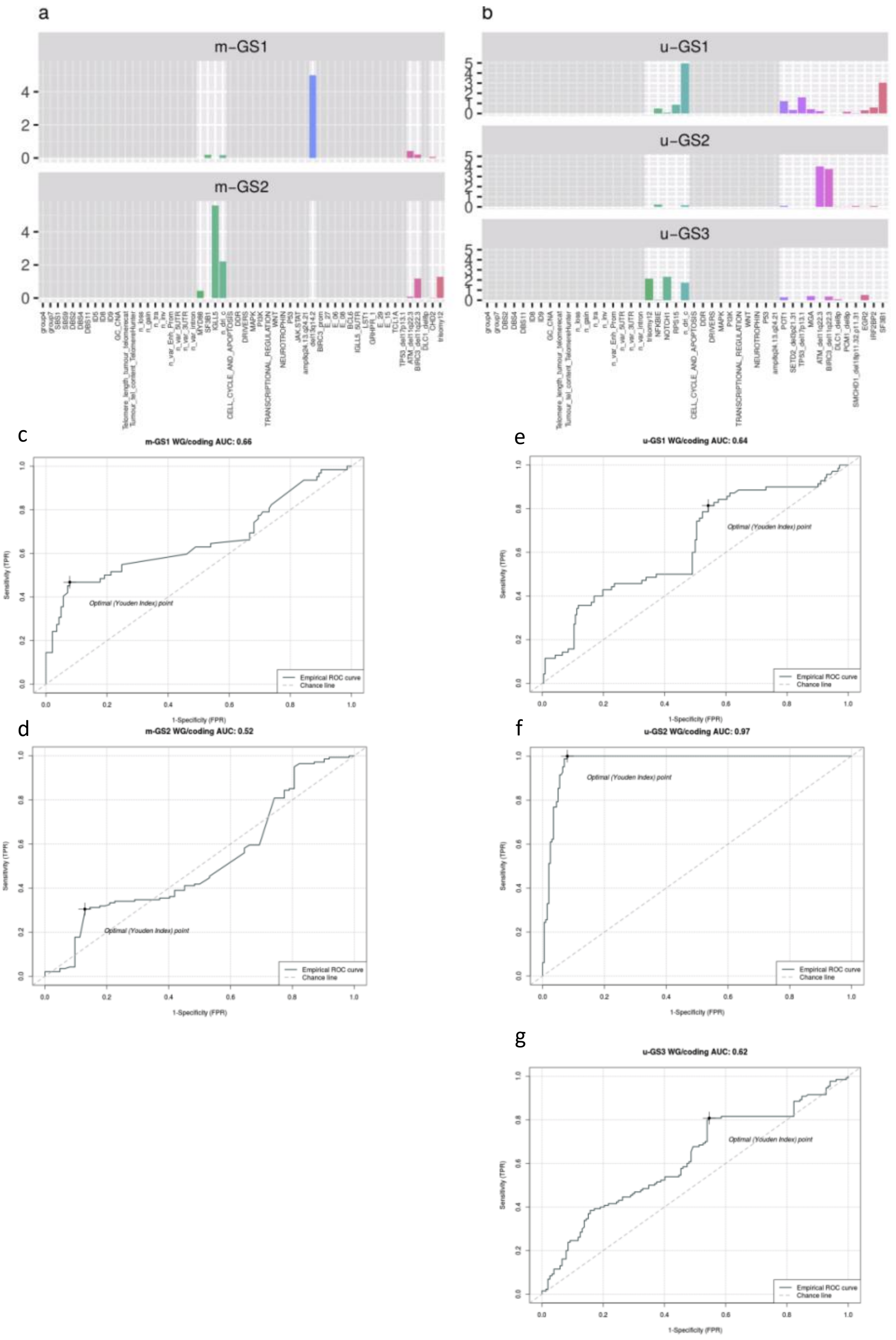

Supplementary figure 7: Genomic subgroup analysis of coding data only compared with WGS data

a-b, Genomic subgroups as derived by coding drivers plus del17p, tri12, del11q and del13q. Grey bars; features available only from WGS data. (a) m-IGHV subgroups, (b) u-IGHV subgroups. **c-g**, Receiver operating characteristic (ROC) curves comparing the signature proportions per sample derived using coding and limited copy-number alterations (CNAs) (del17p, tri12, del11q and del13q) sequence data to the assigned subgroup per sample as derived using WGS. **c-d**, hypermutated subgroups (c) m-GS1; (d) m-GS2. **e-g**, unmutated subgroups (e) u-GS1; (f) u-GS2; (g) u-GS3.

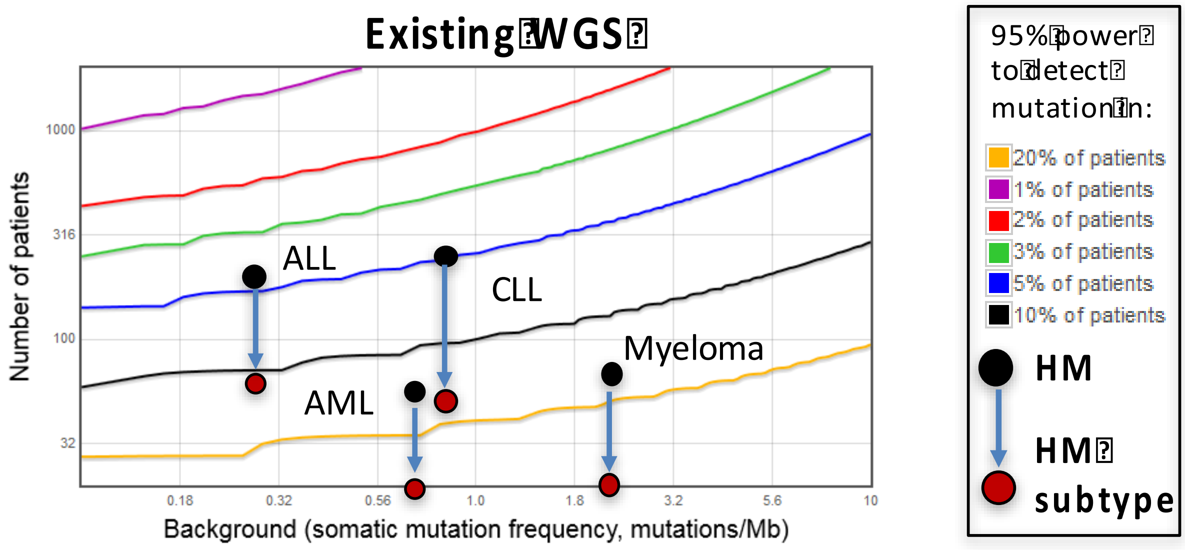

Supplementary figure 8: Power calculations.
To insure the success of this programme, it was critical to have sufficient numbers of samples. WGS data of CLL was publicly available for 234 patients across all different biological and clinical disease subtypes (black dot HM; MBL, CLL, IGHV mutated and unmutated, at diagnosis, treatment naïve, relapsed refractory) meaning that somatic mutations occurring in 5% or more patients had been identified. However, of these 234 patients, only 49 needed treatment. Therefore, possibly, only mutations occurring in 20% or more of these patients had been detected (red dot HM subtype). By increasing the sample size to 450 and selecting exclusively patients who needed treatment, we would able to confidently call mutations occurring at 2% in this subgroup of CLL with 95% power.

References

1. Scrucca, L., Fop, M., Murphy, T. B. & Raftery, A. E. mclust 5: Clustering, Classification and Density Estimation Using Gaussian Finite Mixture Models. *R J.* **8**, 289–317 (2016).
2. Vavoulis, D. V, Cutts, A., Taylor, J. C. & Schuh, A. A statistical approach for tracking clonal dynamics in cancer using longitudinal next-generation sequencing data. *Bioinformatics* **37**, 147–154 (2021).
3. Beekman, R. *et al.* The reference epigenome and regulatory chromatin landscape of chronic lymphocytic leukemia. *Nature Medicine* **24**, 868–880 (2018).
4. Ernst, J. & Kellis, M. Chromatin-state discovery and genome annotation with ChromHMM. *Nat. Protoc.* **12**, 2478–2492 (2017).
5. Rheinbay, E. *et al.* Analyses of non-coding somatic drivers in 2,658 cancer who le genomes. *Nature* **578**, 102–111 (2020).
6. Rao, S. S. P. *et al.* A 3D Map of the Human Genome at Kilobase Resolution Reveals Principles of Chromatin Looping. *Cell* **159**, 1665–1680 (2014).
7. De Paepe, A. Elucidating regulatory elements : studies in chronic lymphocytic leukemia and multiple myeloma. (Karolinska Institutet, 2018).
8. Duttke, S. H., Chang, M. W., Heinz, S. & Benner, C. Identification and dynamic quantification of regulatory elements using total RNA. *Genome Res.* **29**, 1836–1846 (2019).
9. Fishilevich, S. *et al.* GeneHancer: genome-wide integration of enhancers and target genes in

GeneCards. *Database* **2017**, (2017).
10. Ott, C. J. *et al.* Enhancer Architecture and Essential Core Regulatory Circuitry of Chronic Lymphocytic Leukemia. *Cancer Cell* **34**, 982-995.e7 (2018).
11. Lawrence, M. S. *et al.* Mutational heterogeneity in cancer and the search for new cancer-associated genes. *Nature* **499**, 214–8 (2013).
12. Mularoni, L., Sabarinathan, R., Deu-Pons, J., Gonzalez-Perez, A. & López-Bigas, N. OncodriveFML: a general framework to identify coding and non-coding regions with cancer driver mutations. *Genome Biol.* **17**, 128 (2016).
13. Arnedo-Pac, C., Mularoni, L., Muiños, F., Gonzalez-Perez, A. & Lopez-Bigas, N. OncodriveCLUSTL: a sequence-based clustering method to identify cancer drivers. *Bioinformatics* (2019). doi:10.1093/bioinformatics/btz501
14. Martincorena, I. *et al.* Universal Patterns of Selection in Cancer and Somatic Tissues. *Cell* **171**, 1029-1041.e21 (2017).
15. Cingolani, P. *et al.* A program for annotating and predicting the effects of single nucleotide polymorphisms, SnpEff. *Fly (Austin).* **6**, 80–92 (2012).
16. Kircher, M. *et al.* A general framework for estimating the relative pathogenicity of human genetic variants. *Nat. Genet.* **46**, 310–315 (2014).
17. Trifonov, V., Pasqualucci, L., Favera, R. D. & Rabadan, R. MutComFocal: an integrative approach to identifying recurrent and focal genomic alterations in tumor samples. *BMC Syst. Biol.* **7**, 25 (2013).
18. Puente, X. S. *et al.* Non-coding recurrent mutations in chronic lymphocytic leukaemia. *Nature* **526**, 519–524 (2015).
19. Landau, D. A. *et al.* Mutations driving CLL and their evolution in progression and relapse. *Nature* **526**, 525–530 (2015).
20. Stanek, D. *et al.* Prot2HG: a database of protein domains mapped to the human genome. *Database* **2020**, (2020).
21. Weinhold, N., Jacobsen, A., Schultz, N., Sander, C. & Lee, W. Genome-wide analysis of noncoding regulatory mutations in cancer. *Nat. Genet.* **46**, 1160 (2014).
22. Kasar, S. *et al.* Whole-genome sequencing reveals activation-induced cytidine deaminase signatures during indolent chronic lymphocytic leukaemia evolution. *Nat. Commun.* **6**, 8866 (2015).
23. Raudvere, U. *et al.* g:Profiler: a web server for functional enrichment analysis and conversions of gene lists (2019 update). *Nucleic Acids Res.* **47**, W191–W198 (2019).
24. Schwessinger, R. *et al.* DeepC: predicting 3D genome folding using megabase-scale transfer learning. *Nat. Methods* **17**, 1118–1124 (2020).
25. Václav, S., Platoš, J. & Krömer, P. Investigating Boolean Matrix Factorization. *DMMT'08* (2008).
26. Badea, L. Extracting gene expression profiles common to colon and pancreatic adenocarcinoma using simultaneous nonnegative matrix factorization. *Pac. Symp. Biocomput.* 267–78 (2008).
27. Lee, D. D. & Seung, H. S. Algorithms for non-negative matrix factorization. in *14th Annual Neural Information Processing Systems Conference, NIPS 2000 - Denver, CO, United States* (Neural information processing systems foundation, 2001).
28. Brunet, J. P., Tamayo, P., Golub, T. R. & Mesirov, J. P. Metagenes and molecular pattern discovery using matrix factorization. *Proc Natl Acad Sci U S A* **101**, 4164–4169 (2004).
29. Hutchins, L. N., Murphy, S. M., Singh, P. & Graber, J. H. Position-dependent motif characterization using non-negative matrix factorization. *Bioinformatics* **24**, 2684–2690 (2008).
30. Frigyesi, A. & Höglund, M. Non-Negative Matrix Factorization for the Analysis of Complex Gene Expression Data: Identification of Clinically Relevant Tumor Subtypes. *Cancer Inform.* **6**, CIN.S606 (2008).
