# Supplementary material for: Whole-genome sequencing of chronic lymphocytic leukemia identifies subgroups with distinct biological and clinical features
Source: Nat Genet. 2022 Nov 4;54(11):1675–89. doi: 10.1038/s41588-022-01211-y (PMC9649442; doi:10.1038/s41588-022-01211-y)
Supplement: Supplementary file 2 — Reporting Summary [file 41588_2022_1211_MOESM2_ESM.pdf]

nature portfolio

Corresponding author(s): Anna Schuh

Last updated by author(s): Aug 23, 2022

Reporting Summary

Nature Portfolio wishes to improve the reproducibility of the work that we publish. This form provides structure for consistency and transparency in reporting. For further information on Nature Portfolio policies, see our Editorial Policies and the Editorial Policy Checklist.

Statistics

For all statistical analyses, confirm that the following items are present in the figure legend, table legend, main text, or Methods section.

n/a	Confirmed	
☐	☒	The exact sample size (n) for each experimental group/condition, given as a discrete number and unit of measurement
☐	☒	A statement on whether measurements were taken from distinct samples or whether the same sample was measured repeatedly
☐	☒	The statistical test(s) used AND whether they are one- or two-sided<br>*Only common tests should be described solely by name; describe more complex techniques in the Methods section.*
☐	☒	A description of all covariates tested
☐	☒	A description of any assumptions or corrections, such as tests of normality and adjustment for multiple comparisons
☐	☒	A full description of the statistical parameters including central tendency (e.g. means) or other basic estimates (e.g. regression coefficient) AND variation (e.g. standard deviation) or associated estimates of uncertainty (e.g. confidence intervals)
☐	☒	For null hypothesis testing, the test statistic (e.g. F, t, r) with confidence intervals, effect sizes, degrees of freedom and P value noted<br>*Give P values as exact values whenever suitable.*
☒	☐	For Bayesian analysis, information on the choice of priors and Markov chain Monte Carlo settings
☐	☒	For hierarchical and complex designs, identification of the appropriate level for tests and full reporting of outcomes
☐	☒	Estimates of effect sizes (e.g. Cohen's d, Pearson's r), indicating how they were calculated

Our web collection on statistics for biologists contains articles on many of the points above.

Software and code

Policy information about availability of computer code

Data collection	no
Data analysis	BioDiscovery Nexus v10<br>Isaac v03.16.02.19<br>Picard v2.12.1<br>Bwtool1.0<br>Strelka v2.8.4 7<br>Delly1<br>Lumpy 0.2.13<br>Manta 0.28.0<br>Canvas 1.3.1<br>MutSigCV v1.41<br>OncodriveFML v2.2.0<br>OncodriveCLUSTL v1.1.1<br>dNdScv1<br>CovGen v1.0.2<br>SNPeff v4.3<br>survcomp 1.40.0<br>MutComFocal v1.0<br>gprofiler2 v0.2.0<br>Telomere Hunter v1.1.0<br>Telomerecat v1.0<br>Shatterseek v1

deconstructSigs v1.9.0
SigProfilerExtractor v1.0.1810
FactoMineR2.4
glmnet4.1
NMF v0.22.0 R package
mclust V5.4.6, V5.4.9
tidyverse v1.3.0
GenVisR version 1.18.1
DeepHaem v1
IgCaller v1.2.1
AssignSubsets online tool 07.01.22
GenomeStudioV2009.2
Tandem Repeats Finder v4.09
Ensembl VEP GRCh38 release 89.4
MutationMapper from cbioportal accessible from https://www.cbioportal.org/mutation_mapper

For manuscripts utilizing custom algorithms or software that are central to the research but not yet described in published literature, software must be made available to editors and reviewers. We strongly encourage code deposition in a community repository (e.g. GitHub). See the Nature Portfolio guidelines for submitting code & software for further information.

Data

Policy information about availability of data

All manuscripts must include a data availability statement. This statement should provide the following information, where applicable:

- Accession codes, unique identifiers, or web links for publicly available datasets
- A description of any restrictions on data availability
- For clinical datasets or third party data, please ensure that the statement adheres to our policy

All data and analytical tools are available within the Genomics England secure 'Research Environment'. Access is subject to institutional Participation Agreement as per Genomics England guidelines https://www.genomicsengland.co.uk/about-gecip/for-gecip-members/data-and-data-access/.

All DNA sequencing data generated within the study is deposited in the National Genomics Research Library. Data from the National Genomics Research Library, which are held in a secure Research Environment, are available freely to registered users. Please, see https://www.genomicsengland.co.uk/research/academic for more information.
All RNA sequencing data has been deposited in EGA and is available following EGA's data access policy.
The outcome of the clinical studies has been published (See all references in Methods). Access to clinical datasets is subject to data sharing policies of the respective clinical trial units that provided legal sponsorship for the studies and can be made available on request.

Publicly available datasets:
gnomAD v3: https://gnomad.broadinstitute.org/news/2019-10-gnomad-v3-0/
UCSC cytoband table and GeneHancer: downloaded from the UCSC genome browser tables, https://genome.ucsc.edu/cgi-bin/hgTables
GENCODE v29 : Downloaded on the gencode website, https://www.gencodegenes.org/human/release_29.html
All ChIP-seq data and chromatin state segmentation of primary CLL samples: EGA accession number EGAD00001004046.
3D chromatin interactions in GM128786: Under accession number GSE63525 in the Gene Expression Omnibus (GEO)
Prot2HG database freely available at www.prot2hg.com
PAN-CANCER pathway list: https://canopybiosciences.com/product/pancancer-pathways/#panel-details, and listed in supplemental Table 2 in the following publication: https://doi.org/10.1182/blood.2020005650
3D chromatin interactions in one CLL sample : in EGA, under accession number EGAD00001004046.

Field-specific reporting

Please select the one below that is the best fit for your research. If you are not sure, read the appropriate sections before making your selection.

☒ Life sciences ☐ Behavioural & social sciences ☐ Ecological, evolutionary & environmental sciences

For a reference copy of the document with all sections, see nature.com/documents/nr-reporting-summary-flat.pdf

Life sciences study design

All studies must disclose on these points even when the disclosure is negative.

Sample size	The sample size calculation was critical to the success of this programme. Our power calculations considered the heterogeneity of CLL and a background somatic mutation frequency of 0.8 mutations per Mb. This means that in order to reliably detect somatic mutations recurring in 2% of patients with CLL, we need to sequence approximately 500 CLL genomes. No data were excluded from the analyses. The experiments were not randomized. The Investigators were not blinded to allocation during experiments and outcome assessment.
Data exclusions	No data was excluded from the genomics analysis. Analysis including clinical data only included a subset of patients for which clinical outcome data was available.
Replication	not applicable. Each sample was collected from a different patient which totalled in 485 biological replicates. In addition, technical replicates

Replication	were not needed as the sequencing noise and artefacts were controlled for using bioinformatic approaches.
Randomization	not applicable. This study is a non-interventional retrospective and descriptive analysis of the genomic landscape of CLL. Randomization is therefore not required.
Blinding	not applicable. This study is a non-interventional retrospective and descriptive analysis of the genomic landscape of CLL. Blinding is therefore not required.

Behavioural & social sciences study design

All studies must disclose on these points even when the disclosure is negative.

Study description	*Briefly describe the study type including whether data are quantitative, qualitative, or mixed-methods (e.g. qualitative cross-sectional, quantitative experimental, mixed-methods case study).*
Research sample	*State the research sample (e.g. Harvard university undergraduates, villagers in rural India) and provide relevant demographic information (e.g. age, sex) and indicate whether the sample is representative. Provide a rationale for the study sample chosen. For studies involving existing datasets, please describe the dataset and source.*
Sampling strategy	*Describe the sampling procedure (e.g. random, snowball, stratified, convenience). Describe the statistical methods that were used to predetermine sample size OR if no sample-size calculation was performed, describe how sample sizes were chosen and provide a rationale for why these sample sizes are sufficient. For qualitative data, please indicate whether data saturation was considered, and what criteria were used to decide that no further sampling was needed.*
Data collection	*Provide details about the data collection procedure, including the instruments or devices used to record the data (e.g. pen and paper, computer, eye tracker, video or audio equipment) whether anyone was present besides the participant(s) and the researcher, and whether the researcher was blind to experimental condition and/or the study hypothesis during data collection.*
Timing	*Indicate the start and stop dates of data collection. If there is a gap between collection periods, state the dates for each sample cohort.*
Data exclusions	*If no data were excluded from the analyses, state so OR if data were excluded, provide the exact number of exclusions and the rationale behind them, indicating whether exclusion criteria were pre-established.*
Non-participation	*State how many participants dropped out/declined participation and the reason(s) given OR provide response rate OR state that no participants dropped out/declined participation.*
Randomization	*If participants were not allocated into experimental groups, state so OR describe how participants were allocated to groups, and if allocation was not random, describe how covariates were controlled.*

Ecological, evolutionary & environmental sciences study design

All studies must disclose on these points even when the disclosure is negative.

Study description	*Briefly describe the study. For quantitative data include treatment factors and interactions, design structure (e.g. factorial, nested, hierarchical), nature and number of experimental units and replicates.*
Research sample	*Describe the research sample (e.g. a group of tagged Passer domesticus, all Stenocereus thurberi within Organ Pipe Cactus National Monument), and provide a rationale for the sample choice. When relevant, describe the organism taxa, source, sex, age range and any manipulations. State what population the sample is meant to represent when applicable. For studies involving existing datasets, describe the data and its source.*
Sampling strategy	*Note the sampling procedure. Describe the statistical methods that were used to predetermine sample size OR if no sample-size calculation was performed, describe how sample sizes were chosen and provide a rationale for why these sample sizes are sufficient.*
Data collection	*Describe the data collection procedure, including who recorded the data and how.*
Timing and spatial scale	*Indicate the start and stop dates of data collection, noting the frequency and periodicity of sampling and providing a rationale for these choices. If there is a gap between collection periods, state the dates for each sample cohort. Specify the spatial scale from which the data are taken*
Data exclusions	*If no data were excluded from the analyses, state so OR if data were excluded, describe the exclusions and the rationale behind them, indicating whether exclusion criteria were pre-established.*
Reproducibility	*Describe the measures taken to verify the reproducibility of experimental findings. For each experiment, note whether any attempts to repeat the experiment failed OR state that all attempts to repeat the experiment were successful.*
Randomization	*Describe how samples/organisms/participants were allocated into groups. If allocation was not random, describe how covariates were controlled. If this is not relevant to your study, explain why.*

Blinding | *Describe the extent of blinding used during data acquisition and analysis. If blinding was not possible, describe why OR explain why blinding was not relevant to your study.*

Did the study involve field work? ☐ Yes ☐ No

Field work, collection and transport

Field conditions	*Describe the study conditions for field work, providing relevant parameters (e.g. temperature, rainfall).*
Location	*State the location of the sampling or experiment, providing relevant parameters (e.g. latitude and longitude, elevation, water depth).*
Access & import/export	*Describe the efforts you have made to access habitats and to collect and import/export your samples in a responsible manner and in compliance with local, national and international laws, noting any permits that were obtained (give the name of the issuing authority, the date of issue, and any identifying information).*
Disturbance	*Describe any disturbance caused by the study and how it was minimized.*

Reporting for specific materials, systems and methods

We require information from authors about some types of materials, experimental systems and methods used in many studies. Here, indicate whether each material, system or method listed is relevant to your study. If you are not sure if a list item applies to your research, read the appropriate section before selecting a response.

Materials & experimental systems

n/a	Involved in the study	
☒	☐	Antibodies
☒	☐	Eukaryotic cell lines
☒	☐	Palaeontology and archaeology
☒	☐	Animals and other organisms
☐	☒	Human research participants
☐	☒	Clinical data
☒	☐	Dual use research of concern

Methods

n/a	Involved in the study	
☒	☐	ChIP-seq
☒	☐	Flow cytometry
☒	☐	MRI-based neuroimaging

Antibodies

Antibodies used	*Describe all antibodies used in the study; as applicable, provide supplier name, catalog number, clone name, and lot number.*
Validation	*Describe the validation of each primary antibody for the species and application, noting any validation statements on the manufacturer's website, relevant citations, antibody profiles in online databases, or data provided in the manuscript.*

Palaeontology and Archaeology

Specimen provenance	*Provide provenance information for specimens and describe permits that were obtained for the work (including the name of the issuing authority, the date of issue, and any identifying information). Permits should encompass collection and, where applicable, export.*
Specimen deposition	*Indicate where the specimens have been deposited to permit free access by other researchers.*
Dating methods	*If new dates are provided, describe how they were obtained (e.g. collection, storage, sample pretreatment and measurement), where they were obtained (i.e. lab name), the calibration program and the protocol for quality assurance OR state that no new dates are provided.*

☐ Tick this box to confirm that the raw and calibrated dates are available in the paper or in Supplementary Information.

Ethics oversight	*Identify the organization(s) that approved or provided guidance on the study protocol, OR state that no ethical approval or guidance was required and explain why not.*

Note that full information on the approval of the study protocol must also be provided in the manuscript.

Animals and other organisms

Policy information about studies involving animals; ARRIVE guidelines recommended for reporting animal research

Laboratory animals	*For laboratory animals, report species, strain, sex and age OR state that the study did not involve laboratory animals.*
Wild animals	*Provide details on animals observed in or captured in the field; report species, sex and age where possible. Describe how animals were*

Wild animals	*caught and transported and what happened to captive animals after the study (if killed, explain why and describe method; if released, say where and when) OR state that the study did not involve wild animals.*
Field-collected samples	*For laboratory work with field-collected samples, describe all relevant parameters such as housing, maintenance, temperature, photoperiod and end-of-experiment protocol OR state that the study did not involve samples collected from the field.*
Ethics oversight	*Identify the organization(s) that approved or provided guidance on the study protocol, OR state that no ethical approval or guidance was required and explain why not.*

Note that full information on the approval of the study protocol must also be provided in the manuscript.

Human research participants

Policy information about studies involving human research participants

Population characteristics	485 patients with CLL were included in the study. The median age of patients (for those were the data was available) was 65 years old. 74% of patients were males.<br><br>A small subset was enrolled into CLEAR (early stage of the disease, n = 12) and CLL210 (relapse refractory patients, n = 30). All remaining patients were treatment-naïve and in need of treatment according to iwCLL criteria. They were either fit patients receiving frontline treatment with: fludarabine, cyclophosphamide, rituximab (FCR)-based treatment in ARCTIC (n = 61) or AdMIRe (n = 65); or frail patients receiving ofatumumab with either bendamustine or chlorambucil chemo-immunotherapy in RIAltO (n = 92). Patients recruited into FLAIR (n = 225) were randomised to ibrutinib alone or in combination with rituximab or venetoclax or standard first-line FCR treatment.
Recruitment	Patients were recruited into the clinical trials mentioned above and in parallel to the 100,000 Genome Program in accordance to the Declaration of Helsinki Declaration of ethical principles for medical research. Patients therefore could freely decline participation in either of the studies, but still participate in the other. Recruitment occurred in specialist haematology clinics held in over 200 sites across the UK. Patients received written information and were given at least 24hrs, usually more, to reflect on participation and were allowed to withdraw at any point in the study without having to give a reason. Translations of PIS and ICFs were available for patients belonging to any of the major ethnic minorities in the UK. All participants with good quality DNA available for WGS were included in the study.
Ethics oversight	Genomics England Project Ethics and the CLL Pilot ethics (MREC 09/H1306/54)

Note that full information on the approval of the study protocol must also be provided in the manuscript.

Clinical data

Policy information about clinical studies

All manuscripts should comply with the ICMJE guidelines for publication of clinical research and a completed CONSORT checklist must be included with all submissions.

Clinical trial registration	CLEAR (NCT01279252)<br>CLL210 (EudraCT 2010-019575-29)<br>RIAltO (NCT01678430)<br>FLAIR (EudraCT 2013-001944-76)<br>ARCTIC (EudraCT Number:2009-010998-20)<br>AdMIRe (EudraCT number: 2008-006342-25
Study protocol	Full protocols of these clinical studies are considered confidential by the clinical trial units of Leeds and Liverpool Universities and Kings College London<br><br>Details of the clinical trial protocols are published:<br><br>Andrew R. Pettitt et al. Lenalidomide, dexamethasone and alemtuzumab or ofatumumab in<br><br>high-risk chronic lymphocytic leukaemia: final results of the NCRI CLL210 trial. Haematologica<br><br>105, 2868–2871 (2020)<br><br>Howard, D. R. et al. Results of the randomized phase IIB ARCTIC trial of low-dose rituximab in<br><br>previously untreated CLL. Leukemia 31, 2416–2425 (2017).<br><br>Munir, T. et al. Results of the randomized phase IIB ADMIRE trial of FCR with or without<br><br>mitoxantrone in previously untreated CLL. Leukemia 31, 2085–2093 (2017).<br><br>Collett, L. et al. Assessment of ibrutinib plus rituximab in front-line CLL (FLAIR trial): study<br><br>protocol for a phase III randomised controlled trial. Trials 18, 387 (2017).
Data collection	Patients were recruited from over 200 secondary and tertiary haematology referral services across the UK between 2014 and 2018

	Baseline and clinical outcome data were collected prospectively across all sites and underwent source verification and data monitoring by an independent data monitoring committee.
Outcomes	For all trials, the primary endpoint was progression free survival. Secondary endpoints were overall response, safety and overall survival. Outcome measures were assessed according to iwCLL response criteria (Hallek M et al 2008). Safety data was recorded following CTCAE vs 4.0.<br>Outcomes were collected in accordance to internationally agreed definitions for overall response rate, progression free survival, overall survival and minimal residual disease as per Hallek, M. et al. iwCLL guidelines for diagnosis, indications for treatment, response assessment, and supportive management of CLL. Blood 131, 2745–2760 (2018).<br><br>Safety outcomes were defined by CTCAE (for version, see publications of the respective studies).

Dual use research of concern

Policy information about dual use research of concern

Hazards

Could the accidental, deliberate or reckless misuse of agents or technologies generated in the work, or the application of information presented in the manuscript, pose a threat to:

No	Yes	
☐	☐	Public health
☐	☐	National security
☐	☐	Crops and/or livestock
☐	☐	Ecosystems
☐	☐	Any other significant area

Experiments of concern

Does the work involve any of these experiments of concern:

No	Yes	
☐	☐	Demonstrate how to render a vaccine ineffective
☐	☐	Confer resistance to therapeutically useful antibiotics or antiviral agents
☐	☐	Enhance the virulence of a pathogen or render a nonpathogen virulent
☐	☐	Increase transmissibility of a pathogen
☐	☐	Alter the host range of a pathogen
☐	☐	Enable evasion of diagnostic/detection modalities
☐	☐	Enable the weaponization of a biological agent or toxin
☐	☐	Any other potentially harmful combination of experiments and agents

ChIP-seq

Data deposition

☐ Confirm that both raw and final processed data have been deposited in a public database such as GEO.

☐ Confirm that you have deposited or provided access to graph files (e.g. BED files) for the called peaks.

Data access links<br>*May remain private before publication.*	*For "Initial submission" or "Revised version" documents, provide reviewer access links. For your "Final submission" document, provide a link to the deposited data.*
Files in database submission	*Provide a list of all files available in the database submission.*
Genome browser session<br>(e.g. UCSC)	*Provide a link to an anonymized genome browser session for "Initial submission" and "Revised version" documents only, to enable peer review. Write "no longer applicable" for "Final submission" documents.*

Methodology

Replicates	*Describe the experimental replicates, specifying number, type and replicate agreement.*
Sequencing depth	*Describe the sequencing depth for each experiment, providing the total number of reads, uniquely mapped reads, length of reads and whether they were paired- or single-end.*
Antibodies	*Describe the antibodies used for the ChIP-seq experiments; as applicable, provide supplier name, catalog number, clone name, and lot number.*

Peak calling parameters	*Specify the command line program and parameters used for read mapping and peak calling, including the ChIP, control and index files used.*
Data quality	*Describe the methods used to ensure data quality in full detail, including how many peaks are at FDR 5% and above 5-fold enrichment.*
Software	*Describe the software used to collect and analyze the ChIP-seq data. For custom code that has been deposited into a community repository, provide accession details.*

Flow Cytometry

Plots

Confirm that:

☐ The axis labels state the marker and fluorochrome used (e.g. CD4-FITC).

☐ The axis scales are clearly visible. Include numbers along axes only for bottom left plot of group (a 'group' is an analysis of identical markers).

☐ All plots are contour plots with outliers or pseudocolor plots.

☐ A numerical value for number of cells or percentage (with statistics) is provided.

Methodology

Sample preparation	*Describe the sample preparation, detailing the biological source of the cells and any tissue processing steps used.*
Instrument	*Identify the instrument used for data collection, specifying make and model number.*
Software	*Describe the software used to collect and analyze the flow cytometry data. For custom code that has been deposited into a community repository, provide accession details.*
Cell population abundance	*Describe the abundance of the relevant cell populations within post-sort fractions, providing details on the purity of the samples and how it was determined.*
Gating strategy	*Describe the gating strategy used for all relevant experiments, specifying the preliminary FSC/SSC gates of the starting cell population, indicating where boundaries between "positive" and "negative" staining cell populations are defined.*

☐ Tick this box to confirm that a figure exemplifying the gating strategy is provided in the Supplementary Information.

Magnetic resonance imaging

Experimental design

Design type	*Indicate task or resting state; event-related or block design.*
Design specifications	*Specify the number of blocks, trials or experimental units per session and/or subject, and specify the length of each trial or block (if trials are blocked) and interval between trials.*
Behavioral performance measures	*State number and/or type of variables recorded (e.g. correct button press, response time) and what statistics were used to establish that the subjects were performing the task as expected (e.g. mean, range, and/or standard deviation across subjects).*

Acquisition

Imaging type(s)	*Specify: functional, structural, diffusion, perfusion.*
Field strength	*Specify in Tesla*
Sequence & imaging parameters	*Specify the pulse sequence type (gradient echo, spin echo, etc.), imaging type (EPI, spiral, etc.), field of view, matrix size, slice thickness, orientation and TE/TR/flip angle.*
Area of acquisition	*State whether a whole brain scan was used OR define the area of acquisition, describing how the region was determined.*

Diffusion MRI ☐ Used ☐ Not used

Preprocessing

Preprocessing software	*Provide detail on software version and revision number and on specific parameters (model/functions, brain extraction, segmentation, smoothing kernel size, etc.).*
Normalization	*If data were normalized/standardized, describe the approach(es): specify linear or non-linear and define image types used for*

Normalization	*transformation OR indicate that data were not normalized and explain rationale for lack of normalization.*
Normalization template	*Describe the template used for normalization/transformation, specifying subject space or group standardized space (e.g. original Talairach, MNI305, ICBM152) OR indicate that the data were not normalized.*
Noise and artifact removal	*Describe your procedure(s) for artifact and structured noise removal, specifying motion parameters, tissue signals and physiological signals (heart rate, respiration).*
Volume censoring	*Define your software and/or method and criteria for volume censoring, and state the extent of such censoring.*

Statistical modeling & inference

Model type and settings	*Specify type (mass univariate, multivariate, RSA, predictive, etc.) and describe essential details of the model at the first and second levels (e.g. fixed, random or mixed effects; drift or auto-correlation).*
Effect(s) tested	*Define precise effect in terms of the task or stimulus conditions instead of psychological concepts and indicate whether ANOVA or factorial designs were used.*

Specify type of analysis: ☐ Whole brain ☐ ROI-based ☐ Both

Statistic type for inference (See Eklund et al. 2016)	*Specify voxel-wise or cluster-wise and report all relevant parameters for cluster-wise methods.*
Correction	*Describe the type of correction and how it is obtained for multiple comparisons (e.g. FWE, FDR, permutation or Monte Carlo).*

Models & analysis

n/a	Involved in the study
☐	☐ Functional and/or effective connectivity
☐	☐ Graph analysis
☐	☐ Multivariate modeling or predictive analysis

Functional and/or effective connectivity	*Report the measures of dependence used and the model details (e.g. Pearson correlation, partial correlation, mutual information).*
Graph analysis	*Report the dependent variable and connectivity measure, specifying weighted graph or binarized graph, subject- or group-level, and the global and/or node summaries used (e.g. clustering coefficient, efficiency, etc.).*
Multivariate modeling and predictive analysis	*Specify independent variables, features extraction and dimension reduction, model, training and evaluation metrics.*
